# Prevalence of irritable bowel syndrome and its association to mental health among the private university students of Dhaka, Bangladesh

**DOI:** 10.1371/journal.pgph.0004670

**Published:** 2025-05-22

**Authors:** A. B. M. Nahid Hasan, Md. Asaduzzaman, Most. Nourin Mahfuj, Rashedul Islam, Md. Mohasin Kabir Bhuyan, Prosenjit Basak, Azaz Bin Sharif

**Affiliations:** 1 Department of Public Health, North South University, Dhaka, Bangladesh; 2 Public Health Promotion and Development Society (PPDS), Dhaka, Bangladesh; 3 Department of Public Health Nutrition, Primeasia University, Dhaka, Bangladesh; 4 Bangladesh Red Crescent Society, Dhaka, Bangladesh; 5 Department of Philosophy and Social-Political Science, Alexandru Ioan Cuza University of Iasi, Iasi, Romania; 6 Department of Clinical-Surgical Diagnostic and Pediatric Sciences, University of Pavia, Pavia, Italy; 7 Public Health Foundation, Dhaka, Bangladesh; 8 Department of Public Health, University of South Asia, Dhaka, Bangladesh; 9 Global Health Institute, North South University, Dhaka, Bangladesh; McMaster University, CANADA

## Abstract

Irritable Bowel Syndrome is a common gastrointestinal disorder linked to psychological distress and lifestyle factors. In Bangladesh, young adults may experience exacerbated IBS symptoms due to particular hardships. This study aims to evaluate the prevalence of IBS and its association with mental health status among private university students. A cross-sectional study conducted between November 2023 and May 2024 and involved 550 students aged 19–40 years from three private universities in Dhaka, Bangladesh selected through convenience sampling. Data were collected via in-person interviews using a semi-structured questionnaire that included the Rome III and the DASS-21 scale. Pearson’s χ² test was used to explore associations and multiple logistic regression was employed to identify factors influencing IBS. The prevalence of IBS among the students was 31.63%. Female students had higher odds of developing IBS compared to males (AOR = 1.71, 95% CI: 1.19 to 3.36). Psychological distress was strongly associated with IBS, with students experiencing severe anxiety (AOR = 3.14, 95% CI: 1.11 to 7.24) and stress (AOR = 3.39, 95% CI: 1.16 to 6.79) showing increased odds of IBS. Additionally, satisfaction with academic major (AOR = 0.46, 95% CI: 0.18 to 0.96) indicates 54% lower odds of IBS compared to those who were unsatisfied. Physical activity (AOR = 0.64, 95% CI: 0.29 to 0.90) shows a 36% reduction in the odss of IBS with moderate physical activity (20–60 minutes per day). Daily vegetable consumption (AOR = 0.81, 95% CI: 0.67 to 0.89) suggested a 19% lower odds of IBS. Malnutrition (AOR = 1.77, 95% CI: 1.11 to 2.82) was associated with a 77% increased odds of IBS. The findings emphasize the importance of implementing comprehensive campuswide health promotion programs that address psychological distress, promote healthy lifestyle choices, and provide nutritional guidance to alleviate the burden of IBS among them.

## Author summary

Irritable bowel syndrome (IBS) is a common digestive disorder that can significantly impact the quality of life, especially among young adults facing academic and social pressures. This study assessed the prevalence of IBS and its association with mental health and lifestyle factors among private university students in Dhaka, Bangladesh. Findings revealed that approximately one-third of the students were affected by IBS, with females showing a higher likelihood of experiencing the condition. Psychological distress, particularly severe anxiety and stress, was strongly linked to higher odds of IBS. Conversely, satisfaction with academic major, engaging in moderate daily physical activity, and regular vegetable consumption were associated with a reduced risk of IBS. Malnutrition was identified as a significant risk factor, increasing the odds of IBS. The results emphasize the intricate relationship between lifestyle behaviors, nutrition, and mental health in the development of IBS among university students. These findings highlight the importance of campus health programs that support mental well-being, encourage nutritious diets, and promote regular physical activity to help lessen the impact of IBS among students.

## Introduction

Irritable Bowel Syndrome (IBS) is a functional gastrointestinal disorder characterized by chronic abdominal pain, discomfort, and altered bowel habits [1]. In Bangladesh, IBS affects 7.8% of the urban adult population and 6.5% of the rural adult population [2]. Among university students in Bangladesh, the prevalence of IBS is significantly higher (39.3%) [3]. Globally, IBS impacts more than 10% of people, though its prevalence varies by region: 17.5% in Latin America, 9.6% in Asia, 7.1% in North America and Europe, and 5.8% in the Middle East and Africa [4]. Addressing and preventing IBS aligns with the Sustainable Development Goals (SDGs), particularly falls under the goal of promoting good health and well-being [5]. Acknowledging the substantial impact of IBS on quality of life and its association with health conditions is essential for advancing public health efforts [6].

Several factors contribute to the development of IBS, including genetics, diet, lifestyle, and psychological stress [3,7]. University students are particularly vulnerable due to unhealthy eating habits, excessive academic stress, and lifestyle choices such as smoking and alcohol consumption [3,8]. Additionally, physical activity levels and specific nutrition-related variables, such as dietary fiber intake and meal regularity, play crucial roles in the development and management of IBS symptoms [9,10]. Furthermore, the competitive academic environment, financial constraints, and family responsibilities exacerbate IBS symptoms and increase the risk of developing the condition [11,12]. Mental health issues like anxiety, depression, and stress are well-documented contributors to IBS severity and recurrence, as they impact gut health through mechanisms such as the brain-gut axis (GBA) [13].

A substantial proportion of individuals with IBS exhibit elevated trait anxiety and depressive symptoms, and with many fulfilling diagnostic criteria for anxiety disorders [13]. Existing research revealed, 50%–90% of IBS patients exhibit psychiatric disorders, indicating that mental health issues significantly influence both the onset and clinical trajectory of IBS [14]. The GBA plays a crucial role in the persistence of IBS symptoms, reflecting complex interaction between central nervous system, enteric microbiota, and gastrointestinal function [15]. Over the past three decades, researchers have identified numerous dysregulations within the GBA, including altered neuroendocrine signaling and visceral hypersensitivity, which serve as biomarkers and pathophysiological contributors to IBS [16]. University students demonstrate heightened vulnerability due to intersecting stressors—academic demands, career uncertainty, and social dynamics—that exacerbate gastrointestinal dysfunction via GBA-mediated mechanisms [17–19]. Physical inactivity, irregular eating habits, and substance use further increase the risk of IBS. Epidemiological studies indicate that sedentary lifestyles are prevalent among 60%–70% of young adults, significantly correlating with higher IBS incidence [20]. Irregular eating patterns, such as skipping meals or consuming high-fat diets, are reported in 40%–50% of university students, further exacerbating gastrointestinal symptoms [21]. Additionally, substance use, including alcohol and tobacco, is reported in 20%–30% of this demographic, with a strong association between these habits and the severity of IBS symptoms [22]. Furthermore, IBS is linked to multisystem comorbidities such as migraines, fibromyalgia, metabolic dysregulation, and sleep disturbances, culminating in reduced quality of life and social stigmatization [23].

Despite the well-documented global factors contributing to Irritable Bowel Syndrome (IBS), there is a notable lack of comprehensive research on the condition among private university students in Dhaka, Bangladesh. Unlike previous studies that focused on general or specific populations, such as medical students [3], this study specifically targets private university students in Dhaka, a densely populated urban center with unique stressors, including intense academic pressure, distinct lifestyle patterns, and diverse food availability. We aim to address critical gaps in understanding IBS prevalence and its association with mental health within a larger and more representative sample. This study evaluates the status of IBS and its determinants among private university students in Dhaka, Bangladesh.

## Materials and methods

### Ethics statement

The Institutional Review Board (IRB) of the University of South Asia, Bangladesh approved this research protocol (MNFS067/EA23). Prior to collecting data, participants were briefed on the aims of the study. Respondents were assured that the gathered information would solely serve research purposes and that their identities would be kept confidential. All participants provided informed written consent.

### Study design and setting

This cross-sectional study recruited students from three private universities between November 20, 2023, and May 5, 2024. The students were conveniently selected from the North South University (NSU), the Bangladesh University of Health Sciences (BUHS), and the Primeasia University. These institutions were selected to represent a diverse range of students in Dhaka, differing in socioeconomic backgrounds, academic pressure, and student experiences. While students at NSU generally come from more affluent families and often live with their families, BUHS and Primeasia primarily serve students from middle- to lower-middle-class background students, many of whom live independently. Collectively, these institutions enroll over 35,000 students across a wide range of academic disciplines and socio-economic backgrounds [24]. The study population comprised currently enrolled students aged 19–40 years, irrespective of their educational or socioeconomic status. Exclusion criteria included refusal to participate, presence of significant gastrointestinal disorders (e.g., Crohn’s disease, ulcerative colitis), history of major abdominal surgery (e.g., bowel resection), pregnancy, or recent postpartum status at the time of the study.

### Sample Size

The sample size was determined using the Cochran’s formula:


$$n=\frac{z^{2}\;p\;(1-p)}{e^{2}}$$

where:

- *Z *= 1.96 (standard normal variate for a 95% confidence level),
- *p *= 0.392 (prevalence of IBS among university students, derived from prior research) [3],
- *e *= 0.05 (5% margin of error).

Substituting these values yielded an initial sample size of 366. To account for potential non-response, the target sample size was adjusted upward by 10%, resulting in 403 participants. For improved generalizability and representativeness, the research team reached an additional 200 data points, for a total of 603 participants across three universities. After excluding 53 incomplete responses, the final analytical sample comprised 550 participants. This adjustment ensured robustness against attrition while maintaining statistical power for analysis.

### Data collection procedure

Data were collected via in-person interviews utilizing a semi-structured questionnaire which initially developed in English and subsequently translated into Bengali to enhance clarity and comprehension. Participants were approached in various campus settings, including classrooms, cafeterias, and libraries. A pilot study involving 5% of the projected sample size was conducted to validate the questionnaire’s effectiveness. Following feedback from pilot study, the questionnaire was revised to improve consistency and comprehensibility of the study instrument. The pilot study data were excluded from the final analysis to ensure the integrity of the results.

### Variables and measurements

#### Rome III criteria for IBS diagnosis.

The Rome III criteria for diagnosing IBS were meticulously designed to ensure consistent identification across various populations [7]. To classify individuals with and without IBS, a systematic and detailed approach is followed to assess the diagnosis criteria. According to this criteria, a diagnosis of IBS can be made if a person has experienced recurrent abdominal pain or discomfort for at least three days per month in the last three months, with the onset of symptoms occurring at least six months prior to diagnosis. Additionally, the abdominal pain or discomfort must be associated with at least two of the following three features: improvement with defecation, onset associated with a change in the frequency of stool, and onset associated with a change in the form (appearance) of stool [25,26].

#### The DASS-21 scale.

Psychological assessments were conducted using the DASS-21 scale, which measures depression, anxiety, and stress. This scale has been validated, translated, and widely used to measure mental health status in Bangladesh [27,28]. Another recent study also validated the Bangla version of the DASS-21 among Bangladeshi healthcare professionals [29]. Each subscale consists of seven items scored on a four-point Likert scale (0–3), reflecting mental distress over the previous four weeks [30]. Among the psychometric properties of the DASS-21, the scale demonstrated excellent reliability in our sample. The Cronbach’s alpha for the total scale was 0.94, with strong internal consistency for the subscales: Depression (α = 0.88), Anxiety (α = 0.83), and Stress (α = 0.85). These results are consistent with the pilot study, where the overall Cronbach’s alpha was 0.92, further confirming the scale’s reliability. While additional validity analyses (e.g., factor analysis) are planned, the high reliability coefficients and alignment with established psychometric properties of the DASS-21 support its use as a robust tool for assessing depression, anxiety, and stress in our population. Scores for each subscale are summed and doubled, with possible totals ranging from 0 to 42 [30], categorizing severity into five levels: normal, mild, moderate, severe, and extremely severe, as outlined in **Table 1** [30].

**Table 1:** Recommended cut-offs for Depression Anxiety and Stress Scale (DASS-21).

Severity	Depression	Anxiety	Stress
Normal	0-9	0-7	0-14
Mild	10-13	8-9	15-18
Moderate	14-20	10-14	19-25
Severe	21-27	15-19	26-33
Extremely Severe	28+	20+	34+

#### Nutritional status.

To assess nutritional status, the weight (in kilograms) and height (in centimeters) of all participants were carefully measured and recorded, enabling the calculation of Body Mass Index (BMI), a widely utilized and reliable indicator [31]. We classified underweight (BMI < 18.5), overweight (BMI 25.0–29.9), and obese (BMI ≥ 30.0) individuals as malnourished, aligning with the World Health Organization’s definition of malnutrition, which includes undernutrition and overnutrition (overweight/obesity) [32]. This approach is consistent with global practice, as evidenced by a recent study that analyze malnutrition across all BMI categories to capture its multifaceted nature [33]. In addition, the Joint Child Malnutrition Estimates (2023) highlight insufficient progress in addressing overweight prevalence alongside stunting and wasting, while the Global Burden of Disease database reports rising malnutrition burdens among adults across BMI ranges [34].

#### Additional co-variates.

The questionnaire included a comprehensive section designed to collect detailed background information across various domains. Socioeconomic variables include gender, age, educational level, marital status, place of residence (hostel, mess/rent house, family, or relatives), and source of education expenses (family, self, and others) selection and satisfaction with major. Lifestyle and health-related behaviors include smoking habits, physical activity, eating habits (including breakfast and fruit consumption), vegetable intake and sleep duration.

### Statistical analysis

The baseline characteristics of the study participants were described using frequencies and percentages. Pearson’s χ2 tests measured bivariate associations between explanatory variables and IBS status to examine the associations between IBS and various covariates. A multiple logistic regression model was employed to assess the influence of explanatory variables on IBS. For the multivariate modeling, we adjusted for gender, age, educational level, marital status, place of residence, source of educational expenses, major selection, satisfaction with major, smoking habits, physical activity level, breakfast skipping habits, fruit consumption habits, frequency of vegetable intake, sleep duration, and depression, anxiety, and stress status. We chose to include all explanatory variables in the regression model regardless of their significance in the bivariate analysis. As noted by Lo et al., relying solely on statistically significant variables from bivariate analyses can be problematic, as some variables may only reveal their importance when considered within a multivariate context [35].

We assessed multicollinearity among the predictors using the Variance Inflation Factor (VIF). Model fit was evaluated using the Hosmer-Lemeshow goodness-of-fit test. We also examined the model’s discriminatory ability using Receiver Operating Characteristic (ROC) curve analysis and calculated the pseudo R² value. P-values less than 0.05 were considered as the level of significance. All statistical analyses were performed using SPSS software (version 25), and STATA software (version 16).

## Results

Table 2 presents the distribution of IBS status among university students according to their baseline characteristics. Overall, 31.63% of the students were identified as having IBS. More than half of the participants were male (53.09%), and nearly half (49.09%) were aged between 19 and 24 years. The distribution of IBS differed significantly by gender (p = 0.020), with a higher proportion of females reporting IBS compared to males. Approximately one-fourth of the students were married (24.18%), and 58.18% resided in a hostel, mess, or rented house. A small proportion (8.73%) reported that they did not choose their major based on personal preference, while 11.45% were dissatisfied with their chosen major. The distribution of IBS varied notably across these groups, with significant differences observed for both major selection (p < 0.001) and satisfaction with the chosen major (p < 0.001).

**Table 2:** Distribution of IBS Status According to Baseline Characteristics of Respondents.

Variables		Status of IBS		
	n (%)	Normal n(%)	IBS present n(%)	P-value
**Gender**				
Male	292 (53.09)	226 (77.40)	66 (22.60)	**0.020**
Female	258 (46.91)	177 (68.60)	81 (31.40)	
**Age**				
19-24 years	270 (49.09)	196 (72.59)	74 (27.41)	0.376
25-30 years	233 (42.36)	176 (75.54)	57 (24.46)	
>30 years	47 (8.55)	31 (65.96)	16 (34.04)	
**Educational Level**				
Undergraduate	423 (76.91)	318 (75.18)	105 (24.82)	0.065
Graduate	127 (23.09)	85 (66.93)	42 (33.07)	
**Marital Status**				
Married	133 (24.18)	90 (67.67)	43 (32.33)	0.094
Unmarried	417 (75.82)	313 (75.06)	104 (24.94)	
**Place of residence**				
Hostel or mess/rent house	320 (58.18)	238 (74.38)	82 (25.62)	0.491
Family or relatives	230 (41.82)	165 (71.74)	65 (28.26)	
**Source of educational expenses**				
Family	360 (65.45)	259 (71.94)	101 (28.06)	0.333
Self and others	190 (34.55)	114 (75.79)	46 (24.21)	
**Did you choose your major?**				
Yes	502 (91.27)	380 (75.70)	122 (24.30)	**<0.001**
No	48 (8.73)	23 (47.92)	25 (52.08)	
**Are you satisfied with your study?**				
Yes	487 (88.55)	369 (75.77)	118 (24.23)	**<0.001**
No	63 (11.45)	34 (53.97)	29 (46.03)	
**Smoking habit**				
Smoker	109 (19.82)	80 (73.39)	29 (26.91)	0.974
Non-Smoker	441 (80.18)	323 (73.24)	118 (26.76)	
**Physical activity level (per day)**				
<20 min/day	202 (36.73)	131 (64.85)	71 (35.15)	**0.003**
20-60 min/day	258 (46.91)	203 (78.68)	55 (21.32)	
>60 min/day	90 (16.36)	69 (76.67)	21 (23.33)	
**Skip breakfast habit**				
Yes	304 (55.27)	218 (71.71)	86 (28.29)	0.357
No	246 (44.73)	185 (75.20)	61 (24.80)	
**Fruit eating habit**				
Daily	177 (32.18)	144 (81.36)	33 (18.64)	**0.012**
Weekly	276 (50.18)	190 (68.84)	86 (31.16)	
Monthly	97 (17.64)	69 (71.13)	28 (28.87)	
**Frequency of eating adequate vegetables**				
Once a week	346 (62.91)	272 (78.61)	74 (21.39)	**<0.001**
Daily	167 (30.36)	104 (62.28)	63 (37.72)	
Once a month	37 (6.73)	27 (72.97)	10 (27.03)	
**Sleep duration (Per day)**				
4-6 hrs	236 (42.91)	166 (70.34)	70 (29.66)	0.121
7-9 hrs	277 (50.36)	213 (76.90)	64 (23.10)	
>9 hrs	37 (6.73)	24 (64.86)	13 (35.14)	
**Nutritional Status as per BMI**				
Normal	362 (65.82)	276 (76.24)	86 (23.76)	**0.029**
Malnourished	188 (34.18)	127 (67.55)	61 (32.45)	

Regarding lifestyle factors, 36.73% of students reported engaging in less than 20 minutes of daily physical activity, and the distribution of IBS varied significantly across physical activity levels (p = 0.003). Fruit-eating frequency also showed statistically significant differences in IBS distribution (p = 0.012). More than half of the students (55.27%) reported skipping breakfast regularly. The magnitude of IBS fluctuated significantly across vegetable consumption frequencies (p < 0.001), with a higher percentage of IBS cases among students who consumed vegetables less frequently. One-third of the students were classified as malnourished (34.18%) based on BMI, and the distribution of IBS significantly differed by nutritional status (p = 0.029).

Table 3 shows the magnitude of depression, anxiety, and stress levels among the respondents. The mean depression score was 11.63 (± 10.40), with 55.55% experiencing mild to extremely severe depression. The average anxiety score was 10.52 (± 9.07), with 56.73% exhibiting varying degrees of of anxiety. Additionally, 36.55% of respondents reported experiencing some level of stress. The distribution of IBS status differed significantly across all mental health characteristics—depression, anxiety, and stress (p-value < 0.001 in each case).

**Table 3:** Depression, Anxiety & Stress Status of the Respondents.

Variables		Status of IBS		
	n (%)	Normal	IBS present	P-value
**Depression,** Mean ± SD **(*11.63 ± 10.40)***				
Normal	250 (45.45)	198 (79.20)	52 (20.80)	**<0.001**
Mild	80 (14.55)	65 (81.25)	15 (18.75)	
Moderate	129 (23.45)	88 (68.22)	41 (31.78)	
Severe	37 (6.73)	25 (67.57)	12 (32.43)	
Extremely Severe	54 (9.82)	27 (50.00)	27 (50.00)	
**Anxiety,** Mean ± SD **(*10.52 ± 9.07*)**				
Normal	238 (43.27)	196 (82.35)	42 (17.65)	**<0.001**
Mild	49 (8.91)	36 (73.47)	13 (26.53)	
Moderate	119 (21.64)	83 (69.75)	36 (30.25)	
Severe	42 (7.64)	23 (54.76)	19 (45.24)	
Extremely Severe	102 (18.55)	65 (63.73)	37 (36.27)	
**Stress,** Mean ± SD **(*12.64 ± 10.12*)**				
Normal	349 (63.45)	280 (80.23)	69 (19.77)	**<0.001**
Mild	62 (11.27)	45 (72.58)	17 (27.42)	
Moderate	64 (11.64)	41 (64.06)	23 (35.94)	
Severe	54 (9.82)	26 (48.15)	28 (51.85)	
Extremely Severe	21 (3.82)	11 (52.38)	10 (26.73)	

As shown in Table 4, a multiple logistic regression model was constructed to assess the impact of explanatory variables on the IBS after controlling for other variables. The mean VIF for the predictors was 2.11, with no individual predictor exceeding a VIF of 5, indicating no considerable multicollinearity and supporting the stability of the model. The Hosmer-Lemeshow test yielded a p-value of 0.1716, suggesting an adequate model fit with no significant evidence of poor calibration. The pseudo R² value was 0.62, indicating that approximately 62% of the variance in the outcome was explained by the model. Additionally, the ROC curve analysis showed an area under the curve (AUC) of 0.7486, reflecting moderate to good discriminatory power.

**Table 4:** Factors affecting the Irritable Bowel Syndrome among the private university students in Dhaka, Bangladesh.

Variables			95% CI	
	AOR	P- value	LL	UL
**Gender**				
Male (Reference)				
Female	**1.71**	**0.01**	**1.19**	**3.36**
**Age**				
19-24 years	0.56	0.33	0.17	1.79
25-30 years	0.46	0.11	0.18	1.19
>30 years (Reference)				
**Educational Level**				
Undergraduate (Reference)				
Graduate	1.22	0.57	0.60	2.47
**Marital Status**				
Married	1.33	0.31	0.76	2.32
Unmarried (Reference)				
**Place of residence**				
Hostel or mess/rent house (Reference)				
Family or relatives	0.85	0.51	0.53	1.37
**Source of educational expenses**				
Family (Reference)				
Self and others	1.01	0.94	0.60	1.73
**Did you choose your major?**				
Yes	1.05	0.91	0.38	2.88
No (Reference)				
**Are you satisfied with your study?**				
Yes	**0.46**	**0.02**	**0.18**	**0.96**
No (Reference)				
**Smoking habit**				
Smoker (Reference)				
Non-smoker	0.79	0.43	0.45	1.41
**Physical activity level (Per day)**				
<20 min/day (Reference)				
20-60 min/day	**0.64**	**0.03**	**0.29**	**0.90**
>60 min/day	0.61	0.16	0.31	1.22
**Breakfast skipping habit**				
Yes (Reference)				
No	1.00	0.99	0.63	1.58
**Fruit eating Habit**				
Daily	0.85	0.67	0.40	1.78
Weekly	1.58	0.14	0.84	2.95
Monthly (Reference)				
**Frequency of eating adequate vegetables**				
Daily	**0.81**	**0.04**	**0.67**	**0.89**
Weekly	2.22	0.10	0.84	5.84
Monthly (Reference)				
**Sleep duration (Per day)**				
4-6 hrs (Reference)				
7-9 hrs	0.72	0.15	0.45	1.13
>9 hrs	1.25	0.61	0.51	3.01
**Nutritional Status as per BMI**				
Normal (Reference)				
Malnourished	**1.77**	**0.01**	**1.11**	**2.82**
**Depression**				
Normal (Reference)				
Mild	0.52	0.11	0.23	1.16
Moderate	0.91	0.80	0.43	1.91
Severe	0.48	0.19	0.16	1.43
Extremely Severe	1.33	0.62	0.42	4.19
**Anxiety**				
Normal (Reference)				
Mild	1.49	0.30	0.64	3.47
Moderate	1.81	0.10	0.88	3.74
Severe	**3.14**	**0.03**	**1.11**	**7.24**
Extremely Severe	1.51	0.39	0.58	3.95
**Stress**				
Normal (Reference)				
Mild	1.37	0.41	0.64	2.93
Moderate	1.97	0.10	0.86	4.51
Severe	**3.39**	**0.01**	**1.29**	**8.87**
Extremely Severe	1.95	0.34	0.49	7.81

Mental health significantly affects the odds of IBS. Participants with severe anxiety had 3.14 times higher odds of developing IBS compared to those with normal anxiety levels (95% CI: 1.11 to 7.24), while stressed students had 3.39 times higher odds of IBS than their peers with normal stress levels (95% CI: 1.16 to 6.79). Additionally, after controlling for other variables, females had 71% higher odds of having IBS compared to males (95% CI: 1.19 to 3.36). Students satisfied with their major had 54% lower odds of being affected by IBS compared to those who were unsatisfied (95% CI: 0.18 to 0.96). Those engaging in 20–60 minutes of physical activity per day had 36% lower odds of having IBS compared to less physically active groups (95% CI: 0.29 to 0.90). Participants who consumed the recommended amounts of vegetables daily had 19% lower odds of IBS compared to those who consumed vegetables less frequently (95% CI: 0.67 to 0.89). Lastly, malnutrition increased the odds of IBS by approximately 77% (95% CI: 1.11 to 2.82) compare to their healthy counterparts.

## Discussion

This study reveals important findings regarding the prevalence and influencing factors of Irritable Bowel Syndrome (IBS) among private university students in Dhaka, particularly highlighting the notable relationships to mental health and lifestyle factors. To the authors’ knowledge, this is the first study to demonstrate the association between IBS and mental health status among the private university students in Bangladesh. While a previous study conducted in public university located in countryside have explored IBS in relation to dietary habits, lifestyle, and demographic factors, the specific relationship between IBS and mental health indicators such as anxiety, depression, and stress has not been previously assessed in this population [3]. This research used the Rome III criteria to establish a prevalence rate of 31.63%. This prevalence is substantially higher than the general population’s prevalence in Bangladesh, which reported as 6.5% and 7.8% in rural and urban areas, respectively. On the other hand, a study conducted in 2022 among a similar study population found a higher IBS prevalence rate of 39.3% [3]. This difference might be attributed to the timing of the research, which was conducted during the COVID-19 pandemic, a period associated with increased stress and lifestyle changes [36].

The elevated prevalence among students can be attributed to heightened academic stress, irregular dietary habits, and increased psychological distress, all of which are known to exacerbate IBS symptoms [37,38]. Additionally, lifestyle factors such as reduced physical activity, irregular eating schedules, and high caffeine intake, common among students, likely contribute to this higher prevalence. In comparison, studies in other regions have reported varying prevalence rates, underscoring the impact of differing lifestyle, dietary habits, stress levels, and diagnostic methodologies on IBS prevalence [39,40].

The findings demonstrated significantly higher levels of stress (36.55%), anxiety (56.73%), and depression (54.55%) among the private university students. The rise in psychological issues can be attributed to relentless academic pressure, societal expectations, and economic burdens which also compounded by negative media portrayals and political unrest [41–44]. Studies from different parts of the world have also documented substantial psychological distress among university students [45,46]. We found, participants experiencing higher levels of anxiety and stress were more likely to have IBS. This finding corroborates existing literature highlighting the bidirectional relationship between mental health disorders and gastrointestinal symptoms [45,47]. Chronic stress and negative emotional states can exacerbate gastrointestinal symptoms through various physiological mechanisms, including alterations in gut motility, immune function, and GBA signaling [48].

The higher prevalence of IBS among females compared to males, as observed in this study, aligns with global trends [49]. This gender disparity is likely multifactorial, involving a complex interplay of biological, psychological, and sociocultural factors [50]. Hormonal fluctuations, particularly during the menstrual cycle, play a significant role by influencing gastrointestinal (GI) motility, visceral sensitivity, and immune responses, thereby exacerbating IBS symptoms. For instance, oestrogen enhances pain perception, while progesterone may slow gut motility, contributing to symptoms such as constipation [50]. Additionally, females tend to exhibit lower visceral pain thresholds and heightened central nervous system sensitivity, which may further amplify symptom severity [51]. Psychological factors, including stress, anxiety, and depression—which are more prevalent among females—also contribute to this disparity by disrupting the GBAand altering gut function [52]. Furthermore, sociocultural influences, such as healthcare-seeking behaviour and societal expectations, may lead to higher diagnosis rates among females, reflecting broader gender-based differences in symptom reporting and medical consultation [53]. The findings highlighted the importance of nutritional status in gastrointestinal health by showing a significant correlation between university students’ nutritional status and IBS prevalence. Insufficient or excessive consumption of essential nutrients leads malnutrition, which can hinder the digestive system’s efficiency and make people more vulnerable to a range of gastrointestinal conditions, such as IBS [10]. In line with earlier studies, this substantial association shows how poor nutritional status affects gastrointestinal function [23,46]. Changes in gut motility, permeability, and microbiota composition can result from nutritional deficiencies, contributing to IBS pathophysiology [16]. Challenges include academic stress, lack of funds, and irregular dietary habits, which are common among students and can also lead to IBS [54].

According to the results of this research, those who expressed contentment with their academic experiences exhibited notably lower odds of IBS compared to their dissatisfied counterparts. This suggests that positive perceptions of one’s educational journey may act as a protective factor against the development of IBS symptoms. Individuals who feel fulfilled in their lives and studies are likely to experience lower levels of stress and anxiety, which are known triggers for gastrointestinal disorders such as IBS [6]. Conversely, dissatisfaction with academic pursuits may contribute to heightened psychological distress, potentially exacerbating gastrointestinal symptoms [55]. This finding is consistent with other studies that have linked academic satisfaction to lower levels of stress and better overall mental health [20,56].

Another important finding was that participants who engaged in higher levels of physical activity demonstrated lower odds of developing IBS. This aligns with existing literature suggesting the beneficial effects of exercise on gastrointestinal function and symptom management [9,57]. Regular physical activity has been shown to improve gut motility, reduce stress, and enhance overall well-being [9]. Additionally, dietary practices, particularly the consumption of vegetables, were substantially correlated with the prevalence of IBS. Individuals who regularly ate the recommended amount of vegetables had a decreased odds of developing IBS compared to those who only occasionally ate vegetables. This underscores the importance of a balanced diet rich in fiber and nutrients in preventing and managing IBS symptoms [10]. Previous studies have similarly found that a high-fiber diet can alleviate IBS symptoms by improving bowel regularity and reducing gastrointestinal discomfort [6,58].

## Strengths and limitations

### Strengths

This study has several strengths. First, the is the first research to investigate the prevalence of Irritable Bowel Syndrome (IBS) and its association with mental health among the private university students in Bangladesh. Despite using a convenient sampling method, the inclusion of institutions with diverse student populations strengthens the representativeness and depth of the data. The use of the Rome III criteria for IBS diagnosis ensures consistency and comparability with other studies, while the DASS-21 scale provides a validated and reliable measure of mental health status. A relatively large sample size (n = 550) and robust statistical analyses, enhance the reliability and generalizability of the findings.

### Limitations

Despite its strengths, this study has some limitations. First, the cross-sectional design limits the ability to establish causal relationships between IBS and its associated factors. Longitudinal studies are needed to better understand the temporal relationships and potential mechanisms underlying these associations. Second, the use of convenience sampling may introduce selection bias, as participants were recruited from only three universities in Dhaka, which may not fully represent the broader student population in Bangladesh. Third, self-reported data on dietary habits, physical activity, and mental health may be subject to recall bias or social desirability bias. Lastly, this study is the use of WHO BMI cut-offs instead of Asian-specific BMI cut-offs, which may affect the generalizability of findings to the Bangladeshi population. Future studies could incorporate objective measures, such as biomarkers or clinical assessments, to complement self-reported data.

### Implications and future directions

The findings of this study have several implications for clinical practice, public health interventions, and future research. Healthcare professionals working with university students should consider the impact of demographic characteristics, lifestyle habits, and mental health on the development and management of IBS. Public health interventions aimed at promoting healthy lifestyle habits, reducing stress, and improving mental well-being may help mitigate the burden of IBS among university students. Implementing regular counseling sessions in universities can proactively address and manage stress, anxiety, and depression among students. Future research should focus on longitudinal studies to elucidate the causal relationships between lifestyle factors, mental health, and IBS development, as well as interventions aimed at reducing the prevalence and impact of IBS in this population.

## Conclusions

The findings of this research reveal that approximately one-third of participants are affected by Irritable Bowel Syndrome (IBS). The study underscores a strong association between IBS and mental health issues, particularly anxiety and stress, as well as lifestyle factors such as physical activity, vegetable consumption, and malnutrition. Female students were found to be at higher risk, while satisfaction with academic major emerged as a protective factor. These findings highlight the need for integrated interventions that address both mental health and lifestyle factors to mitigate the burden of IBS among university students. By raising awareness and implementing targeted health promotion programs, universities can play a vital role in improving the gastrointestinal and overall well-being of students in Bangladesh and similar settings.

## Supporting information

S1 Data Dataset used in this research. (XLS)

S1 Text Questionnaire used to measure IBS. (PDF)
