# Supplementary material for: Prevalence of irritable bowel syndrome and its association to mental health among the private university students of Dhaka, Bangladesh
Source: PLOS Glob Public Health. 2025 May 22;5(5):e0004670. doi: 10.1371/journal.pgph.0004670 (PMC12097621; doi:10.1371/journal.pgph.0004670)
Supplement: S1 Text — (PDF) [file pgph.0004670.s002.pdf]

Details measure questionnaire for IBS of the participants

ROME III questionnaire:

1. In the last 3 months, how often did you have discomfort or pain anywhere in your abdomen?

    - Never
    - < One day/month
    - One day/month
    - Two-three days/month
    - One day/week
    - One day/week
    - Everyday

2. Only for women: Did this discomfort or pain occur only during your menstrual bleeding and not at other times?

    - No
    - Yes

3. Have you had this discomfort or pain 6 months or longer?

    - No
    - Yes

4. How often did this discomfort or pain get better or stop after you had a bowel movement?

    - Never or rarely
    - Sometimes
    - Often
    - Most of the time
    - Always

5. When this discomfort or pain started, did you have more frequent bowel movements?

    - Never or rarely
    - Sometimes
    - Often
    - Most of the time
    - Always

6. When this discomfort or pain started, did you have less frequent bowel movements?

    - Never or rarely
    - Sometimes
    - Often
    - Most of the time

- Always

7. When this discomfort or pain started, were your stools (bowel movements) looser?

- ➢ Never or rarely
- ➢ Sometimes
- ➢ Often
- ➢ Most of the time
- ➢ Always

8. When this discomfort or pain started, how often did you have harder stools?

- ➢ Never or rarely
- ➢ Sometimes
- ➢ Often
- ➢ Most of the time
- ➢ Always

9. In the last 3 months, how often did you have hard or lumpy stools?

- ➢ Never or rarely
- ➢ Sometimes
- ➢ Often
- ➢ Most of the time
- ➢ Always

10. In the last 3 months, how often did you have loose, mushy or watery stools?

- ➢ Never or rarely
- ➢ Sometimes
- ➢ Often
- ➢ Most of the time
- ➢ Always
